# Comparison of the effect of DLI according to cell sources in relapsed AML after allogeneic stem cell transplantation

**DOI:** 10.1007/s00277-023-05093-w

**Published:** 2023-01-20

**Authors:** Woochan Park, Ja Min Byun, Junshik Hong, Inho Kim, Dong-Yeop Shin, Seonyang Park, Youngil Koh, Sung-Soo Yoon

**Affiliations:** 1 Department of Internal Medicine, Seoul National University Hospital, 101 Daehak-Ro, Jongno-Gu, Seoul, 03080 Republic of Korea; 2 Cancer Research Institute, Seoul National University College of Medicine, Seoul, Republic of Korea; 3 Department of Internal Medicine, Haeundae Paik Hospital, Inje University College of Medicine, Busan, Republic of Korea

**Keywords:** Acute myeloid leukemia, Relapse, Donor lymphocyte infusion, G-CSF, Salvage

## Abstract

For relapsed acute myeloid leukemia (AML) patients who received allogeneic hematopoietic stem cell transplantation, donor lymphocyte infusion (DLI) is an effective therapy. However, the cell source of DLI remains a topic of debate. In this study, we aimed to compare the efficacy and safety of G-CSF mobilized cells (G-DLI) with conventionally collected DLI (C-DLI). A total of 81 patients (50 C-DLI vs. 31 G-DLI) were assessed for clinical outcomes. There were no statistically significant differences in the baseline characteristics between the two groups including AML risk, donor types, interval from relapse to DLI, and infused CD3^+^ cell count. Although not statistically significant, complete remission (CR) and chimerism conversion rates were higher in G-DLI than in C-DLI: 51.6% vs. 28.0%, *P* = 0.057 and 42.3% vs. 28.2%, *P* = 0.363, respectively. There was no difference in acute graft-versus-host disease (GVHD) incidence and severity of acute GVHD between the two groups. The median overall survival (OS) of the G-DLI and C-DLI groups was 139 days and 106 days, respectively (*P* = 0.58). In conclusion, G-DLI appears to be a safe and an equally efficacious substitute for C-DLI, which is more readily available.

**Supplementary Information:**

The online version contains supplementary material available at 10.1007/s00277-023-05093-w.

## Introduction

Allogeneic hematopoietic stem cell transplantation (alloSCT) represents a curative treatment option for many acute myeloid leukemia (AML) patients. Unfortunately, relapse remains the main cause of treatment failure and impedes the survival of patients. The optimal treatment strategy for relapse-after-alloSCT setting remains to be determined; donor lymphocyte infusion (DLI) offers an attractive option. Based on the theoretical graft-versus-leukemia (GVL) effect mediated by donor T-cells, DLI was first applied to patients who experienced a relapse after alloSCT to treat chronic myeloid leukemia in 1990. With its success, the field of DLI flourished and many patients who had the choices of either only going through a second transplant or to succumbing to their underlying hematologic disease are being saved [1–4].

There are, however, some unresolved issues and areas for improvements. In general, DLI involves the re-collection of peripheral blood lymphocytes from the initial donor. The main hurdle for this conventional donor lymphocyte infusion (C-DLI) is the availability and safety of the donor, as unexpected adverse events can occur upon repeated cycles of collection. The development of graft-versus-host disease (GVHD) and aplasia also poses a problem [5, 6]. Such concerns have led to an interest in using the excess cryopreserved cells collected from the first alloSCT as the source of granulocyte-colony stimulating factor (G-CSF) mobilized DLI. G-CSF mobilized DLI (G-DLI) is especially appealing as previous studies have shown that G-CSF mobilized peripheral blood progenitor cell infusion leads to superior disease-free survival without increasing GVHD rates in relapsed patients [7, 8]. Recognizing the insufficiency of data on the efficacy and safety of G-DLI in comparison to C-DLI, particularly in Asian patients, we carried out this study to determine whether G-DLI can be a safe substitute for C-DLI.

## Methods

### Patients

This is a retrospective longitudinal cohort study conducted between January 2001 and December 2017 at the Seoul National University Hospital. AML patients aged 16 years or older and undergoing therapeutic DLI for the first time due to a relapse after alloSCT were included in this study. Preemptive DLI, prophylactic DLI, and DLI for the treatment of engraftment failure were excluded. We included patients with morphologic relapse or extramedullary relapse in our analysis.

Their medical records were reviewed and analyzed for demographics, baseline disease characteristics, details of alloSCT, information and outcomes of DLI, and survival. The data on CD3^+^ cell count of the donor lymphocytes and CD3^+^/CD34^+^ cell count of the residual stem cells were also collected. This study was conducted according to the Declaration of Helsinki and was approved by the institutional review board of Seoul National University Hospital (IRB No. H-1903–020-1015). All authors had access to the study data and reviewed and approved this study.

### Definitions

AML diagnosis was made according to the classification criteria used at the respective time of histological diagnosis [9]. The European LeukemiaNet classification was used for cytogenetic subgrouping [10], and since most patients were diagnosed before the next-generation sequencing era, only cytogenetic results by G-banding and fluorescence in situ hybridization (FISH) were considered for risk stratification. Treatment response and relapse were evaluated per the International Working Group guidelines [11]. Patients who survived for more than two years were defined as long-term survivors [12].

A chimerism study was performed by analyzing short tandem repeats (STRs) using PCR amplification [13]. Complete chimerism (CC) was defined as a complete changeover to donor cells. The coexistence of donor and recipient cells (1–99%) was defined as mixed chimerism (MC). When there were no donor cells found, the term “recipient cell only” was used. The chimerism conversion was defined as the change from MC or recipient cell only to CC after DLI.

Both newly developed acute GVHD after DLI and deterioration of known GVHD after DLI were documented. Acute GVHD grading was performed according to the Mount Sinai Acute GVHD International Consortium (MAGIC) standard criteria [14]. Fatal GVHD was defined as the death of a patient from uncontrolled GVHD. Chronic GVHD was classified as mild, moderate, or severe according to the 2014 National Institutes of Health consensus criteria [15].

### DLI

In this study, C-DLI was defined as the infusion of lymphocytes additionally collected from an unstimulated donor who was the previous alloSCT donor, whereas G-DLI was defined as the infusion of remnant stem cells that were cryopreserved after the initial alloSCT without prophylactic immunosuppression conditioning [4]. Patients with remnant stem cells cryopreserved after previous alloSCT received G-DLI. The remaining peripheral blood stem cells from the initial alloSCT were cryopreserved − 190 °C, and these were the cell sources of G-DLI. Cryopreserved peripheral blood stem cells were thawed at 37 °C prior to infusion. The salvage chemotherapy for the relapsed patient was used per the attending physician’s decision. And patients with a history of immunosuppressant use before one month of relapse were included in the analysis.

### Statistical analysis

Statistics differences between groups were assessed by using Student’s *t* test or one-way analysis of variance for continuous variables, and Pearson’s chi-square test for categorical variables. Survival analysis was performed using the Kaplan–Meier method. Overall survival (OS) was defined as the time from the first DLI to death from any cause. If patients survived without confirmed death or progression, survival was censored at the latest date of follow-up; data available up to July 2019 were used. Univariate and multivariate proportional hazards regression models were used to identify independent risk factors for OS by using Cox proportional hazard models. A stepwise backward procedure was used to construct a set of independent predictors at each endpoint. All predictors with *P* value below 0.10 were considered, and sequentially removed if the *P* value in the multiple model was above 0.05. All data were analyzed using R version 3.6.0 (The R foundation, Vienna, Austria). *P* values of < 0.05 were considered statistically significant.

## Results

### Patient characteristics

As shown in Table 1 and between the C-DLI and G-DLI groups, there were no significant differences in the baseline characteristics including AML risk, alloSCT setting, used conditioning regimen, and status at DLI. Most patients (93.8%) received the salvage chemotherapy before DLI, and the details of chemotherapy used are summarized in Supplemental Table 1. Chemotherapies commonly used for induction were used in relapsed patients, and among them, standard-dose cytarabine with idarubicin was the most used (43.2%). Of the 76 patients who received chemotherapy before DLI, 66 patients underwent DLI without evaluation after chemotherapy, and 10 patients were evaluated for disease status before DLI. Nine patients underwent bone marrow examination, of which 4 patients had persistent disease, 3 patients had inconclusive for remission because of low cellularity, and 2 patients obtained bone marrow remission. One patient was assessed with chimerism study and had complete chimerism. Of the patients who did not receive salvage chemotherapy, 4 received DLI immediately with early relapse, and one received DLI with extramedullary relapse.

**Table 1:** Baseline characteristics

Characteristics	All	C-DLI	G-DLI	*P*
	(*N* = 81)	(*N* = 50)	(*N* = 31)	
Median age, years (range)^1^	45.6 (16.2–65.5)	43.8 (16.2–65.5)	46.9 (16.4–62.9)	0.096
Male sex, *n* (%)	36 (44.4%)	25 (50%)	11 (35.5%)	0.201
Cytogenetic risk, *n* (%)				0.823
Low	13 (16.0%)	9 (18.0%)	4 (12.9%)	
Intermediate	46 (56.8%)	28 (56.0%)	18 (58.1%)	
High	22 (27.2%)	13 (26.0%)	9 (29.0%)	
Graft source				0.157
Bone marrow	9 (11.1%)	8 (16.0%)	1 (3.2%)	
Peripheral blood stem cell	72 (88.9%)	42 (84.0%)	30 (96.8%)	
Conditioning regimen, *n* (%)				0.684
MAC	23 (28.4%)	15 (30.0%)	8 (25.8%)	
NMC or RIC	58 (71.6%)	35 (70.0%)	23 (74.2%)	
Donor type, *n* (%)				0.841
Sibling, FM	53 (65.4%)	34 (68.0%)	19 (61.3%)	
Unrelated, FM	16 (19.8%)	10 (20.0%)	6 (19.4%)	
Unrelated, PM	6 (7.4%)	3 (6.0%)	3 (9.7%)	
Haploidentical	6 (7.4%)	3 (6.0%)	3 (9.7%)	
Status at alloSCT, *n* (%)				0.052
CR1	42 (51.9%)	21 (42.0%)	21 (67.7%)	
CR > 1	17 (21.0%)	14 (28.0%)	3 (9.7%)	
Non-CR	22 (27.2%)	15 (30.0%)	7 (22.6%)	
Chimerism at DLI, *n* (%)				0.268
Recipient cell only	3 (3.7%)	3 (6.0%)	0 (0%)	
Mixed chimerism	67 (82.7%)	42 (84.0%)	25 (80.6)	
Complete chimerism	6 (7.4%)	2 (4.0%)	4 (12.9%)	
Missing	5 (6.2%)	3 (6.0%)	2 (6.5%)	
Recipient DNA, %, median (range)	50.3 (0.0–100)	61.8 (0.0–100)	45.7 (0.0–91.2)	0.34
Interval from alloSCT to relapse, days, median (range)	156 (14.0–4960)	153 (14.0–4960)	161 (18.0–1030)	0.370
Interval from relapse to DLI, days, median (range)	26 (2.0–202)	28.5 (2.0–202)	21.0 (5.0–199)	0.588
GVHD at DLI^2^	18 (22.2%)	11 (22.0%)	7 (22.6%)	0.951
ISA before relapse, *n* (%)				0.791
Cyclosporin	52 (64.2%)	32 (64.0%)	20 (64.5%)	
Tacrolimus	6 (7.4%)	3 (6.0%)	3 (9.7%)	
No use	23 (28.4%)	15 (30.0%)	8 (25.8%)	
Chemotherapy before DLI, *n* (%)	76 (93.8%)	47 (94.0%)	29 (93.5%)	0.935

^1^Age at diagnosis

^2^Includes both chronic and acute graft-versus-host disease

*C-DLI*, conventional donor lymphocyte infusion; *G-DLI*, G-CSF mobilized lymphocyte infusion; *MAC*, myeloablative conditioning; *NMC*, non-myeloablative conditioning; *RIC*, reduced intensity conditioning; *FM,* fully HLA-matched donor; *PM*, partially HLA-matched donor; *alloSCT*, allogeneic stem cell transplantation; *CR1*, first complete remission; CR > 1, second or more complete remission; *non-CR*, non-complete remission; *GVHD*, graft-versus-host disease; *ISA*, immunosuppressant; *DLI*, donor lymphocyte infusion

Over 80% of the patients underwent alloSCT from full-matched donors and 72.9% of the patients were in complete remission (CR) at the time of alloSCT. Among the partially matched unrelated donors, there were three 9 of 10 HLA-matched donors in the C-DLI group, two 9 of 10 HLA-matched donors in the G-DLI group, and one 8 of 10 HLA-matched donor in the G-DLI group. Among the patients who underwent alloSCT from haploidentical donors, one patient of the C-DLI group received busulfan–cyclophosphamide–antithymocyte globulin conditioning, and the remaining 5 patients received busulfan–fludarabine–antithymocyte globulin conditioning. There were 2 patients who underwent alloSCT despite harboring favorable cytogenetic risk: one did not achieve CR after second induction and the other progressed during consolidation. At the time of DLI, the majority of the patients (82.7%) showed mixed chimerism, and 22.2% of the patients had active GVHD. Among the 6 patients with CC, 5 underwent DLI due to extramedullary relapse and 1 showed increased blast count (8%) on bone marrow examination. About 70% of patients were using ISA one month before relapse, but all discontinued after relapse was confirmed.

### Outcomes of DLI

There was no difference in the number of cells infused as shown in Table 2. The median CD3^+^ cell count for C-DLI and G-DLI groups were 1.044 × 10^8^/kg and 0.855 × 10^8^/kg, respectively (*P* = 0.356). The patients with the maximum (6.9 × 10^8^/kg) and minimum (0.1 × 10^8^/kg) number of CD3^+^ cell counts were both in the C-DLI group. The median CD34^+^ cell count for the G-DLI group was 2.34 × 10^6^/kg. There was no significant difference between the CD3^+^ cell counts used in 22 DLIs conducted before 2011 and 56 DLIs conducted thereafter. (0.76 × 10^8^/kg and 1.06 × 10^8^/kg, respectively, *P* = 0.181).

**Table 2:** Clinical outcomes of donor lymphocyte infusion

Characteristics	All	C-DLI	G-DLI	*P*
	(*N* = 81)	(*N* = 50)	(*N* = 31)	
CD3^+^ cells in DLI, × 10^8^/kg (range) *	0.974 (0.1–6.9)	1.044 (0.1–6.9)	0.855 (0.18–2.2)	0.356
CD34^+^ cells in DLI, × 10^6^/kg (range)	-	-	2.340 (0.1–6.42)	
Complete remission, *n (*%)	30 (37.0%)	14 (28.0%)	16 (51.6%)	0.057
Chimerism conversion, *n (*%)**	22 (33.8%)	11 (28.2%)	11 (42.3%)	0.363
Newly developed or aggravated GVHD after DLI, *n (*%)	35 (43.2%)	22 (44.0%)	13 (41.9%)	0.855
Acute GVHD grading, *n* (%)				0.23
Grade I	12 (14.8%)	8 (16.0%)	4 (12.9%)	
Grade II	10 (12.3%)	4 (8.0%)	6 (19.4%)	
Grade III	3 (3.7%)	3 (6.0%)	0 (0%)	
Grade IV	10 (12.3%)	7 (14.0%)	3 (9.7%)	
Fatal GVHD, *n* (%)	6 (7.4%)	4 (8.0%)	2 (6.5%)	0.414
Persistent aplasia, *n* (%)	23 (28.4%)	13 (26.0%)	10 (32.3%)	0.724
Median neutropenic period, days (range)***	9 (3–90)	9 (3–90)	8 (3–36)	0.228
Median overall survival, days (range)	115 (3–2933)	106 (3–2476)	139 (8–2933)	0.58

*C-DLI*, conventional donor lymphocyte infusion; *G-DLI*, G-CSF mobilized lymphocyte infusion; *CR*, complete remission; *GVHD*, graft-versus-host disease; *DLI*, donor lymphocyte infusion

^*^There are 3 missing values for CD3^+^ cell counts

^**^Data on chimerism conversion after DLI was available from 65 patients (39 C-DLI and 26 G-DLI)

^***^The neutropenic period is defined as from DLI to the first day of three consecutive days of peripheral blood neutrophil count of > 500 × 10^6^/L

Among the 81 patients assessed, 30 (37%) achieved bone marrow remission after cell therapy. G-DLI showed a trend towards increased CR rate (51.6% vs. 28%); however, difference did not reach statistical significance (*P* = 0.057). Data on chimerism conversion after DLI were available from 65 patients (39 C-DLI and 26 G-DLI). A higher chimerism conversion rate was noted in the G-DLI group (11/26, 42.3%) than in C-DLI (11/39, 28.2%), but there was no statistically significant difference (*P* = 0.363). Among 3 patients who had recipient cells only chimerism at chimerism analyses, 2 patients died without chimerism study, and one patient got chimerism conversion after DLI. The one who got chimerism conversion survived over 300 days after DLI. One patient did not recover from bone marrow aplasia and died of pneumonia on day 13 from DLI. The other patient recovered from neutropenia 5 days after DLI, but blasts began to appear again in the peripheral blood a month later. He received two additional chemotherapy treatments and two more DLIs, but died from intracranial hemorrhage 180 days after the initial DLI.

There was no difference in the number of patients with persistent aplasia between the two groups(26.0%, 32.3%, respectively, *P* = 0.724). Among patients with persistent aplasia after DLI, the mean value of percentages of recipient cells in patients with mixed chimerism before DLI did not show significant difference between two groups (65.4% and 54.4%, respectively, *P* = 0.463). And, there was no difference in time from DLI to the first day of three consecutive days of peripheral blood neutrophil count of > 500 × 10^6^/L (9 days, 8 days, respectively, *P* = 0.228).

**Fig. 1:**
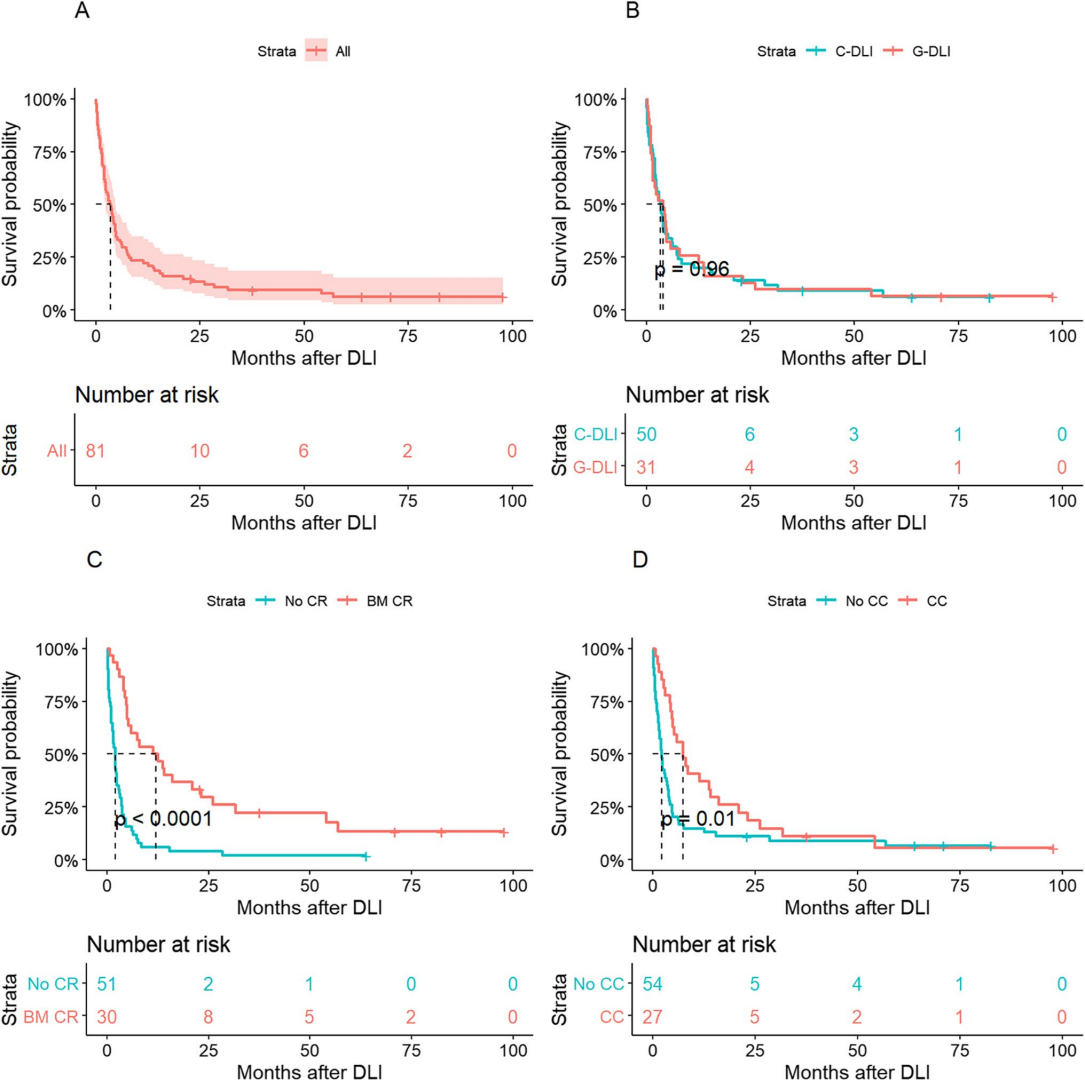
Survival outcomes of 81 patients subjected to donor lymphocyte infusion (DLI). (**a**) All patients. (**b**) Overall survival (OS) according to DLI source. (**c**) OS according to bone marrow remission achievement after DLI. (**d**) OS according to chimerism conversion status after DLI

The median overall survival after DLI was 3.5 months (range, 0.1–56.9 months) for the entire group (Table 2 and Fig. 1a). There were no differences in 1-year OS rates between the C-DLI and G-DLI groups: 20.0% and 22.6%, respectively (Fig. 1b). Patients who achieved bone marrow remission and/or chimerism conversion after DLI presented better survival than those who did not. More specifically, the median OS of patients who achieved bone marrow remission was 12 months compared to 2 months in those who did not (*P* < 0.001*,* Fig. 1c). As for chimerism conversion, patients who achieved CC after DLI showed significantly longer survival compared to those who did not (median 7.4 months vs. 2.1 months, respectively, *P* = 0.01) as shown in Fig. 1d. From multivariate analyses (Table 3), persistent disease after the first DLI (bone marrow remission vs. persistent, HR 4.85, 95% CI 2.40–9.80, *P* < 0.001), alloSCT donor type (matched sibling donor vs. haplo-identical donor, HR 2.68, 95% CI 1.10–6.48, *P* = 0.036), and shorter interval from transplantation to relapse (over 5 months vs. within 5 months, HR 1.68, 95% CI 1.03–2.74, *P* = 0.037) were considered as prognostic factors of OS. Whether DLI was performed once or additional treatment was not a good prognostic factor, which is because the response to treatment and the additional treatment were heterogenous. Among the patients who received additional treatment in the C-DLI group, 12 patients received additional chemotherapy and DLI, 5 patients received secondary transplantation. In the G-DLI group, 15 patients received additional chemotherapy and DLI, 2 patients received secondary transplantation.

**Table 3:** Univariate and multivariate analyses for overall survival

	Univariate analysis		Multivariate analysis	
	HR (95% CI)	*P*	HR (95% CI)	*P*
Cell source				
C-DLI	1			
G-DLI	0.97 (0.61–1.54)	0.881		
Cytogenetic risk				
Good	1		1	
Intermediate	1.23 (0.63–2.38)	0.551	0.96 (0.48–1.93)	0.920
Poor	2.09 (1.00–4.38)	0.049	1.93 (0.92–4.05)	0.083
Treatment				
DLI once only	1			
Additional treatment	0.76 (0.48–1.21)	0.244		
Donor				
Sibling, FM	1		1	
Unrelated, FM	0.83 (0.45–1.54)	0.555	1.14 (0.59–2.20)	0.457
Unrelated, PM	0.54 (0.21–1.37)	0.193	0.51 (0.20–1.34)	0.294
Haploidentical	3.47 (1.45–8.30)	0.005	2.68 (1.10–6.48)	0.036
Bone marrow status after first DLI				
CR	1		1	
Persistent	3.64 (2.20–6.04)	< 0.001	4.85 (2.40–9.80)	< 0.001
Chimerisms after first DLI				
Complete chimerism	1		1	
Others	1.85 (1.14–3.02)	0.013	0.94 (0.48–1.82)	0.850
GVHD after DLI				
Newly developed or aggravated	1			
None	1.33 (0.84–2.12)	0.227		
Interval from alloSCT to relapse				
> 5 months	1		1	
< 5 months	1.53 (0.97–2.42)	0.069	1.68 (1.03–2.74)	0.037

*C-DLI*, conventional donor lymphocyte infusion; *G-DLI*, G-CSF mobilized lymphocyte infusion; *DLI*, donor lymphocyte infusion; *FM*, fully HLA-matched donor; *PM*, partially HLA-matched donor; *CR*, complete remission; *GVHD*, graft-versus-host disease; *alloSCT*, allogeneic stem cell transplantation

### GVHD

Among the patients, 35 (43.2%) showed signs of acute GVHD after DLI (Table 2), but there was no significant difference in the incidence rates between the C-DLI and G-DLI groups. Among them, 18 patients experienced aggravation of pre-existing GVHD. Of the 11 patients in the C-DLI group who had GVHD before DLI, 8 patients experienced aggravation of GVHD (72.7%). In the G-DLI group, 4 out of 7 patients (57.1%) experienced exacerbation of GVHD. There was a trend towards the increased grades III-IV acute GVHD in the C-DLI group (20%) compared with the G-DLI group (9.7%), but a similar incidence of fatal GVHD between the 2 groups (8.0% vs. 6.5%, respectively, *P* = 0.414). Although there were differences between patients, the main treatment for acute GVHD after DLI was the use of steroids and ISA.

### Long-term survivors

There were 10 patients who were classified as long-term survivors (patients who survived longer than 2 years after DLI), among whom 6 and 4 underwent C-DLI and G-DLI, respectively. Table 4 shows details about these 10 patients. All 10 patients received salvage chemotherapy before DLI. There were 5 patients who remained alive at the time of data collection. There were 2 patients (patients 3 and 6) who had long remission duration but ultimately experienced central nervous system relapse and died. Three patients (patients 4, 8, and 9) underwent a second alloSCT from another donor after achieving bone marrow remission with DLI. Patient 4 and patient 8 maintained bone marrow remission and received a second alloSCT from a full matched unrelated donor. Patient 9 underwent second alloSCT due to CNS relapse 5 months after DLI. In the case of patient 7, the first bone marrow examination after DLI showed an slight increase in blast cells, which was suspected of being persistent disease. However, as a result of follow-up bone marrow examination without any additional treatment, bone marrow remission was determined.

**Table 4:** Long-term survivors

Patient number, age, sex	Cytogenetic risk	Disease state at alloSCT	Donor specifics	AlloSCT to relapse/relapse to DLI (days)	Type/DLI once or additional treatment	CD3^+^ cell count (× 10^8^/kg)/CD34^+^ cell count (× 10^6^/kg)	Response to DLI (bone marrow)	Chimerism	Acute GVHD	OS (days)	Status at last follow-up
1, 16/F	Intermediate	CR1	Unrelated, FM	100/83	G-DLI/once	NA/6.35	CR	CC	Present	2933	Alive
2. 36/F	Good	CR2	Sibling, FM	278/24	C-DLI/additional	1.27/NA	CR	MC	None	2476	Alive
3. 40/F	Good	CR1	Sibling, FM	466/199	G-DLI/additional	0.8/1.16	CR	MC	Present	2126	Expired
4. 33/M	Intermediate	CR1	Unrelated, PM	265/43	C-DLI/additional	0.43/NA	CR	MC	Present	1916	Alive
5. 16/F	Intermediate	CR2	Unrelated, PM	197/28	C-DLI/once	0.7/NA	CR	MC	Present	1708	Expired
6. 43/M	Intermediate	CR1	Sibling, FM	684/37	G-DLI/additional	1.3/2.63	CR	CC	None	1624	Expired
7. 33/F	Intermediate	CR4	Unrelated, FM	414/181	C-DLI/once	0.74/NA	Persistent (delayed CR)	CC	Present	1129	Alive
8. 46/M	Poor	CR1	Sibling, FM	51/19	C-DLI/additional	0.38/NA	CR	CC	Present	951	Expired
9. 20/M	Intermediate	CR1	Sibling, FM	4960/41	C-DLI/ additional	0.13/NA	Persistent	MC	None	900	Expired
10. 40/M	Poor	Induction failure	Sibling, FM	170/55	G-DLI/once	0.49/3.73	CR	CC	None	835	Alive

*alloSCT*, allogeneic stem cell transplantation; *DLI*, donor lymphocyte infusion; *GVHD*, graft-versus-host disease; *OS*, overall survival; *CR*, complete remission; *FM*, fully HLA-matched donor; *PM*, partially HLA-matched donor; *C-DLI*, conventional donor lymphocyte infusion; *G-DLI*, G-CSF mobilized lymphocyte infusion; *NA*, not available; *CC*, complete chimerism; *MC*, mixed chimerism

## Discussion

The importance of this study are that (1) as reported, we showed that DLI is a viable salvage option for AML patients relapsed after alloSCT, (2) G-CSF mobilized DLI yields a prognosis similar to that of conventional DLI, and (3) cryopreservation of graft content does not negatively affect the outcomes of DLI. In fact, G-DLI showed trends towards better CR rates and chimerism conversion rates compared to C-DLI without increasing GVHD incidence. Since G-DLI uses remnant stem cells collected from a previous alloSCT, there is no need for donor revalidation and additional collection, which also thereby provides time and economic benefits. Since this study did not consider the duration of salvage chemotherapy before DLI, it was not possible to see the difference in consumption time according to the two cell sources. Therefore, additional studies are needed to see if there is a difference in preparation time until DLI is administered between the two groups. Moreover, the G-DLI utilized in our study were cryopreserved and all the C-DLI were fresh. With no formal study on the impact of graft content cryopreservation on clinical outcomes, we hereby provide real-world evidence that it is safe to use cryopreserved products for later treatment.

G-CSF induces the mobilization of dendritic cells in humans and subsequently makes the dendritic cells available in peripheral lymphoid organs. This leads to the presentation of self-peptides and Th2 polarization in the donor [16, 17]. Since donor Th2 cells are less likely to induce aGVHD, the mobilization by G-CSF have been associated with reduced severity of aGVHD [16, 18, 19]. In line with such findings, patients who underwent G-DLI showed less grades III-IV aGVHD after DLI (Table 2). When dealing with patients relapsing after an alloSCT, attending physicians and patients alike may suffer from heavy emotional and physical burden of GVHD occurrence. In this regard, G-DLI may have certain advantages. Importantly, attenuated GVHD severity does not mean that G-CSF mobilized grafts lose their GVL effects. In fact, a different mechanism mainly concerning the perforin-dependent pathway [20] is responsible for GVL; thus, G-CSF mobilized grafts are able to retain their GVL effects while benefiting from decreased GVHD severity. Likewise, we observed similar, if not superior, CR or CC rates between G-DLI and C-DLI. In this study, patients treated with both kinds of DLI showed short duration of neutropenia compared to previous study that treating relapsed AML patients with chemotherapy followed by G-CSF primed DLI [7]. However, there was no significant difference between the C-DLI group and G-DLI group. This suggests that the presence of stem cells in the DLI product and the period of neutropenia may not be related. Further controlled studies are required.

As previously reported [21–23], DLI proved to be a promising salvage option with 1-year survival rates of over 20% (22.6% and 20.0% for the G-DLI and C-DLI groups, respectively). Several factors such as the achievement of hematological remission at DLI, favorable cytogenetics, and a longer duration of remission after alloSCT have been associated with good prognosis after DLI [24, 25]. Likewise, we recognized a longer interval from alloSCT to relapse and bone marrow remission after DLI as good prognostic factors (Table 3). Although alloSCT with haplo-identical donors was observed as a poor prognostic factor, careful interpretation is warranted. All of the alloSCT from haplo-donors was carried out prior to 2010; thus, these patients did not benefit from the advances in GVHD prophylaxis regimens including post-cyclophosphamide and improved infection control methodologies. Considering the fact that 4 of the 6 haplo-cases died due to GVHD and 2 due to infection within 60 days of DLI, this finding warrants further investigations. We wanted to ensure that patients received alloSCT and DLI from haplo-identical donors did not act as a bias. Therefore, baseline characteristics were analyzed between the two groups except for the six patients, and survival analysis was additionally performed. As a result, there was no significant difference from that of the entire patient group. The results are presented in supplemental Table 2 and supplemental Fig. 1.

In this study, 6 patients had CC at the baseline chimerism study. One of them had AML relapse while maintaining CC. The patient’s initial alloSCT donor was a matched sibling donor. It appears that some genetic abnormalites from donor cell can trigger the development of donor cell origin AML. After reinduction chemotherapy and the first DLI, the patient received chemotherapy and DLI once more and achieved bone marrow CR. However, cord compression occurred due to chloroma in the spine, and after surgery, the patient died of pneumonia. The remaining five patients with CC had extramedullary relapses. The treatment of extramedullary relapses has not yet been established. The DLI is considered as one of the treatment options [26, 27]. However, in sites where immune escape is possible, the GVL effect is known to be diminished, which would result in the less efficacy of the DLI in these sites [28].

One of the most obvious limitations of this study is its retrospective nature. The patients who will undergo C-DLI or G-DLI were not classified according to the certain criteria, but were analyzed retrospectively. Furthermore, although 81 cases of DLI are not trivial for a single center, it may perhaps be a little small to draw statistically powerful conclusions. Additionally, for G-DLI, the cryopreserved CD34^+^ cell count was not based on thawing and remeasurement, but was estimated based on the amount collected during the initial alloSCT. As for the limitations in terms of the effectiveness of DLI, especially in patients who did not have bone marrow remission or complete chimerism after DLI, the median overall survival was very short, about 2 months. As a result of this, in the case of patients who do not obtain remission with DLI, additional treatment should be considered as soon as possible. On the other hand, most of the patients in this study received salvage chemotherapy before DLI. In most cases, DLI is rapidly followed after chemotherapy, so hematological remission status cannot be assessed in the meantime. Lastly, since the duration of the study period spans from 2001 to 2017, many aspects of acute leukemia diagnosis and treatment, including risk stratification, use of target therapies, and supportive care, have altered over the course of time. However, we believe these shortcomings do not diminish the importance of our findings that can be readily incorporated into clinical practice.

In conclusion, we provide evidence that G-DLI is a readily available option that is worth considering for AML patients who have had a relapse after alloSCT. Further investigations on optimizing DLI use should ensue, but in the absence of established guidelines, this study provides physicians with better insights into clinical experience with DLI use.

## Supplementary Information

Supplemental Fig. 1 Survival outcomes of 75 patients subjected to donor lymphocyte infusion (DLI) except haploidentical donors. (a) All patients, The median overall survival was 3.8 months (range, 0.1–56.9 months). (b) Overall survival (OS) according to DLI source. The median survival between 2 groups showed no significant differences (3.5 months vs. 4.5 months, P = 0.78). (c) OS according to bone marrow remission achievement after DLI; the median OS of patients who achieved bone marrow remission was 12.6 months compared to 2.05 months in those who did not (P < 0.001). (d) OS according to chimerism conversion status after DLI. The patients who achieved CC after DLI showed significantly longer survival compared to those who did not (median 7.7 months vs. 2.3 months, respectively, P = 0.014) (PNG 129 kb)

High Resolution Image (TIF 11554 kb)

ESM 1 (DOCX 21 kb)

## Data Availability

Data sharing not applicable to this article as no datasets were generated or analyzed during the current study.

## Code availability

This is not applicable.
