# Supplementary figures and images for: Comparison of the effect of DLI according to cell sources in relapsed AML after allogeneic stem cell transplantation
Source: Ann Hematol. 2023 Jan 20;102(3):629–39. doi: 10.1007/s00277-023-05093-w (PMC9977844; doi:10.1007/s00277-023-05093-w)

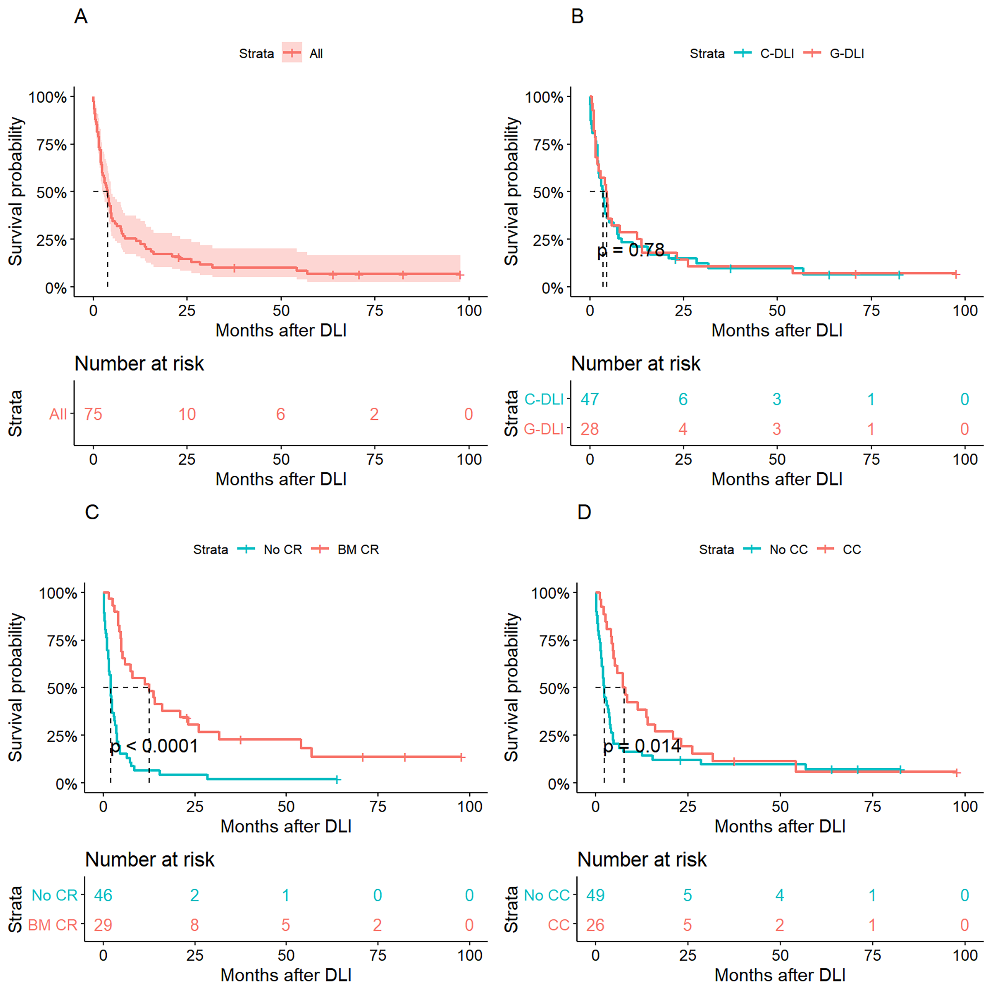

Supplement: Supplementary file 1 — Survival outcomes of 75 patients subjected to donor lymphocyte infusion (DLI) except haploidentical donors. (a) All patients, The median overall survival was 3.8 months (range, 0.1–56.9 months). (b) Overall survival (OS) according to DLI source. The median survival between 2 groups showed no significant differences (3.5 months vs. 4.5 months, P = 0.78). (c) OS according to bone marrow remission achievement after DLI; the median OS of patients who achieved bone marrow remission was 12.6 months compared to 2.05 months in those who did not (P < 0.001). (d) OS according to chimerism conversion status after DLI. The patients who achieved CC after DLI showed significantly longer survival compared to those who did not (median 7.7 months vs. 2.3 months, respectively, P = 0.014) (PNG 129 kb) [file 277_2023_5093_Fig2_ESM.png]

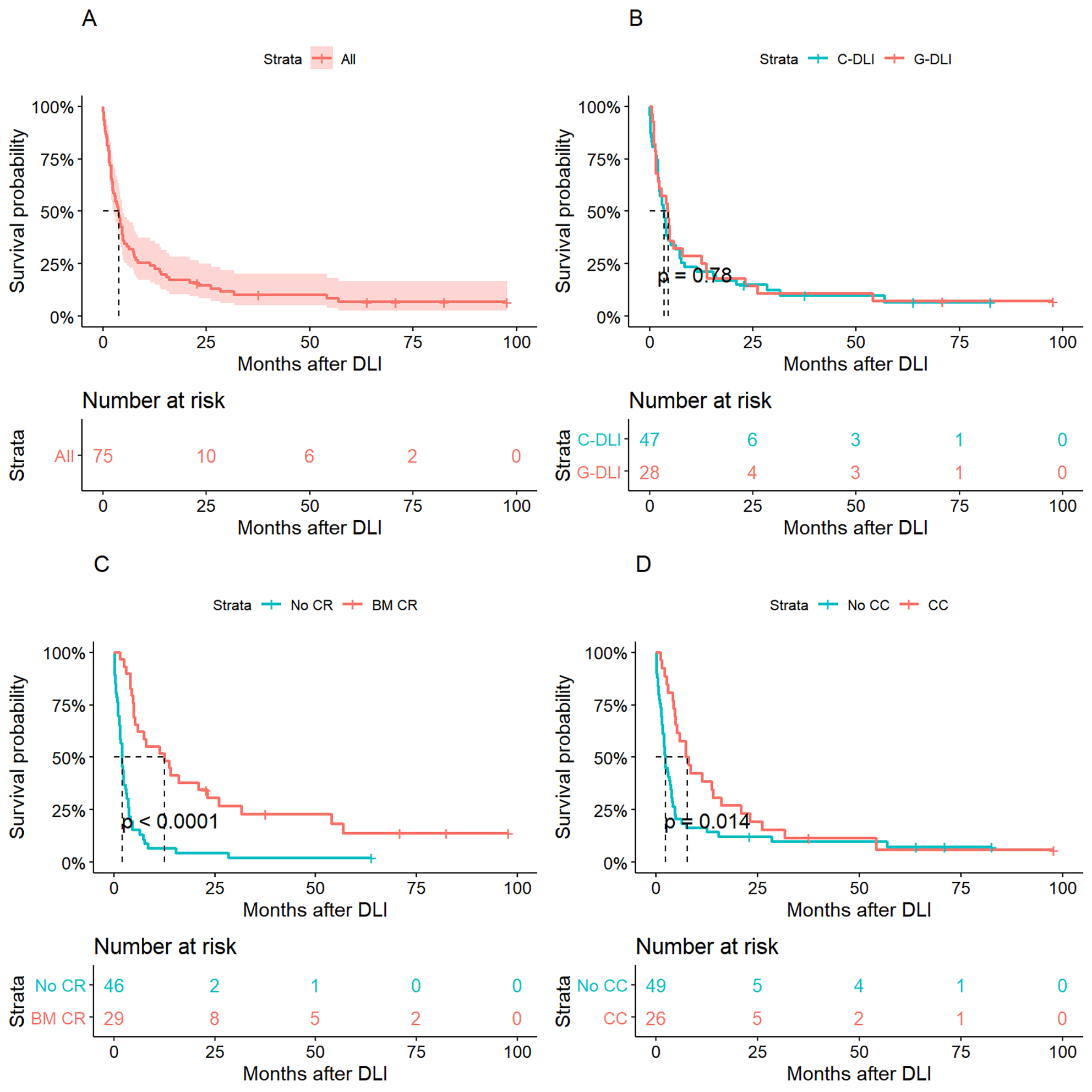

Supplement: Supplementary file 2 — High Resolution Image (TIF 11554 kb) [file 277_2023_5093_MOESM2_ESM.tif]
