# Supplementary material for: Comparison of the effect of DLI according to cell sources in relapsed AML after allogeneic stem cell transplantation
Source: Ann Hematol. 2023 Jan 20;102(3):629–39. doi: 10.1007/s00277-023-05093-w (PMC9977844; doi:10.1007/s00277-023-05093-w)
Supplement: Supplementary file 3 — (DOCX 21 kb) [file 277_2023_5093_MOESM3_ESM.docx]

Supplemental table 1. Salvage chemotherapy regimen

| Chemotherapy regimen | All | C-DLI | G-DLI |
| --- | --- | --- | --- |
| (*N*, %) | (*N* = 81) | (*N* = 50) | (*N*= 31) |
| IA | 35 (43.2%) | 25 (50.0%) | 10 (32.3%) |
| HiDAC | 10 (12.3%) | 7 (14.0%) | 3 (9.7%) |
| MA | 9 (11.1%) | 4 (8.0%) | 5 (16.1%) |
| ME | 3 (3.7%) | 2 (4.0%) | 1 (3.2%) |
| AEP | 3 (3.7%) | 1 (2.0%) | 2 (6.5%) |
| FLAG-I | 9 (11.1%) | 4 (8.0%) | 5 (16.1%) |
| CLAG-M | 7 (8.6%) | 4 (8.0%) | 3 (9.7%) |
| No chemotherapy | 5 (6.2%) | 3 (6.0%) | 2 (6.5%) |

*IA*, standard-dose cytarabine with idarubicin; *HiDAC*, high-dose cytarabine; *MA*, standard-dose cytarabine plus mitoxantrone; *ME*, mitoxantrone and etoposide; *AEP*, epirubicin, cisplatin, and etoposide; *FLAG-I*, fludarabine plus cytarabine, G-CSF, and idarubicin; *CLAG-M*, cladribine, cytarabine, G-CSF, and mitoxantrone.

Supplemental table 2. Baseline characteristics except haploidentical donors

| Characteristics | All | C-DLI | G-DLI | *P* |
| --- | --- | --- | --- | --- |
|  | (*N* = 75) | (*N* = 47) | (*N*= 28) |  |
| Median age, years (range)^1^ | 45.6 (16.2–65.5) | 44.7 (16.2–65.5) | 46.3 (16.4–62.9) | 0.193 |
| Male sex, *n* (%) | 34 (45.3%) | 23 (48.9%) | 11 (39.3%) | 0.567 |
| Cytogenetic risk, *n* (%) |  |  |  | 0.74 |
| Low | 13 (17.3%) | 9 (19.1%) | 4 (14.3%) |  |
| Intermediate | 44 (58.7%) | 26 (55.3%) | 18 (64.3%) |  |
| High | 18 (24.0%) | 12 (25.5%) | 6 (21.4%) |  |
| Graft source, *n* (%) |  |  |  | 0.25 |
| Bone marrow | 8 (10.7%) | 7 (14.9%) | 1 (3.6%) |  |
| Peripheral blood stem cell | 67 (89.3%) | 40 (85.1%) | 27 (96.4%) |  |
| Conditioning regimen, *n* (%) |  |  |  | 0.964 |
| MAC | 23 (30.7%) | 15 (31.9%) | 8 (28.6%) |  |
| NMC or RIC | 52 (69.3%) | 32 (68.1%) | 20 (71.4%) |  |
| Donor type, n (%) |  |  |  | 0.794 |
| Sibling, FM | 53 (70.7%) | 34 (72.3%) | 19 (67.9%) |  |
| Unrelated, FM | 16 (21.3%) | 10 (21.3%) | 6 (21.4%) |  |
| Unrelated, PM | 6 (8.0%) | 3 (6.4%) | 3 (10.7%) |  |
| Status at alloSCT, *n* (%) |  |  |  | 0.157 |
| CR1 | 39 (52.0%) | 21 (44.7%) | 18 (64.3%) |  |
| CR > 1 | 16 (21.3%) | 13 (27.7%) | 3 (10.7%) |  |
| Non-CR | 20 (26.7%) | 13 (27.7%) | 7 (25.0%) |  |
| Chimerism at DLI, *n* (%) |  |  |  | 0.508 |
| Recipient cell only | 2 (2.7%) | 2 (4.3%) | 0 (0%) |  |
| Mixed chimerism | 63 (84.0%) | 40 (85.1%) | 23 (82.1) |  |
| Complete chimerism | 5 (6.7%) | 2 (4.3%) | 3 (10.7%) |  |
| Missing | 5 (6.7%) | 3 (6.4%) | 2 (7.1%) |  |
| Recipient DNA, %, median (range) | 47.1 (0.0–100) | 53.5 (0.0–100) | 45.7 (0.0–91.2) | 0.701 |
| Interval from alloSCT to relapse, days, median (range) | 156 (14.0–4960) | 152 (14.0–4960) | 159 (18.0–840) | 0.265 |
| Interval from relapse to DLI, days, median (range) | 26 (2.0–202) | 28.0 (2.0–202) | 25.5 (5.0–199) | 0.718 |
| GVHD at DLI^2^ | 15 (20.0%) | 10 (21.3%) | 5 (17.9%) | 0.952 |
| ISA before relapse, *n* (%) |  |  |  | 0.791 |
| Cyclosporin | 50 (66.7%) | 31 (66.0%) | 19 (67.9%) |  |
| Tacrolimus | 3 (4.0%) | 1 (2.1%) | 2 (7.1%) |  |
| No use | 22 (29.3%) | 15 (31.9%) | 7 (25.0%) |  |
| Chemotherapy before DLI, *n* (%) | 71 (94.7%) | 44 (93.6%) | 27 (96.4%) | 1 |

^1^Age at diagnosis

^2^Includes both chronic and acute graft-versus-host disease

*C-DLI*, conventional donor lymphocyte infusion; *G-DLI*, G-CSF mobilized lymphocyte infusion; *MAC*, myeloablative conditioning; *NMC*, non-myeloablative conditioning; *RIC*, reduced intensity conditioning; *FM*, fully HLA-matched donor; *PM*, partially HLA-matched donor; *alloSCT*, allogeneic stem cell transplantation; *CR1*, first complete remission; CR > 1, second or more complete remission; *non-CR*, non-complete remission; *GVHD*, graft-versus-host disease; *ISA*, immunosuppressant; *DLI*, donor lymphocyte infusion
